# Mutations in the SLC2A9 Gene Cause Hyperuricosuria and Hyperuricemia in the Dog

**DOI:** 10.1371/journal.pgen.1000246

**Published:** 2008-11-07

**Authors:** Danika Bannasch, Noa Safra, Amy Young, Nili Karmi, R. S. Schaible, G. V. Ling

**Affiliations:** 1 Department of Population Health and Reproduction, School of Veterinary Medicine, University of California Davis, Davis, California, United States of America; 2 Department of Veterinary Clinical Sciences, School of Veterinary Medicine, Purdue University, West Lafayette, Indiana, United States of America; 3 Department of Medicine and Epidemiology, School of Veterinary Medicine, University of California Davis, Davis, California, United States of America; Stanford University School of Medicine, United States of America

## Abstract

Allantoin is the end product of purine catabolism in all mammals except humans, great apes, and one breed of dog, the Dalmatian. Humans and Dalmatian dogs produce uric acid during purine degradation, which leads to elevated levels of uric acid in blood and urine and can result in significant diseases in both species. The defect in Dalmatians results from inefficient transport of uric acid in both the liver and renal proximal tubules. Hyperuricosuria and hyperuricemia (huu) is a simple autosomal recessive trait for which all Dalmatian dogs are homozygous. Therefore, in order to map the locus, an interbreed backcross was used. Linkage mapping localized the huu trait to CFA03, which excluded the obvious urate transporter 1 gene, SLC22A12. Positional cloning placed the locus in a minimal interval of 2.5 Mb with a LOD score of 17.45. A critical interval of 333 kb containing only four genes was homozygous in all Dalmatians. Sequence and expression analyses of the SLC2A9 gene indicated three possible mutations, a missense mutation (G616T;C188F) and two promoter mutations that together appear to reduce the expression levels of one of the isoforms. The missense mutation is associated with hyperuricosuria in the Dalmatian, while the promoter SNPs occur in other unaffected breeds of dog. Verification of the causative nature of these changes was obtained when hyperuricosuric dogs from several other breeds were found to possess the same combination of mutations as found in the Dalmatian. The Dalmatian dog model of hyperuricosuria and hyperuricemia underscores the importance of SLC2A9 for uric acid transport in mammals.

## Author Summary

Animals excrete waste products in their urine. When most mammals metabolize compounds, called purines, they produce allantoin as one waste product in their urine. Humans, great apes, and Dalmatian dogs produce a different breakdown product, uric acid. This leads to high levels of uric acid in the urine and blood. In humans, this can result in diseases such as kidney stones and gout and may cause hypertension. In Dalmatians, high uric acid levels result in bladder stones that often have to be removed surgically. The cause of high uric acid levels in humans and great apes is not the same as in the Dalmatian dog. Here we report the genetic cause of the Dalmatian condition. This change is shared by dogs from unrelated breeds, indicating that it predates the separation of dog breeds. The gene that causes excretion of uric acid in Dalmatians is important for controlling the amount of uric acid in human blood and is therefore important for human diseases. It is not clear why humans and great apes have evolved to excrete uric acid, but it appears that some dogs have developed a different mechanism that leads to the same result: elevations in urine and blood uric acid levels.

## Introduction

Uric acid is the predominant product of purine metabolism in humans, great apes and one breed of dog, the Dalmatian; all other mammals excrete allantoin. During primate evolution, urate oxidase (UOX), which catalyzes the oxidation of uric acid into allantoin, accumulated several independent nonsense mutations that led to its silencing and resulted in high serum and urine uric acid levels in humans and great apes [1],[2]. Huu in the Dalmatian results from a different cause [3],[4]. Uric acid freely circulates in the form of urate, the salt of uric acid, in the plasma where it serves as a free-radical scavenger. Although uric acid has evolved in humans to be the main product of purine metabolism, this change has had some negative effects. High levels of urate predispose humans to gout [5],[6]. In addition, uric acid levels have been correlated with hypertension, vascular disease and metabolic syndrome although it is unclear whether hyperuricemia is primary or secondary in these cases [7]–[10].

**Figure 1:**
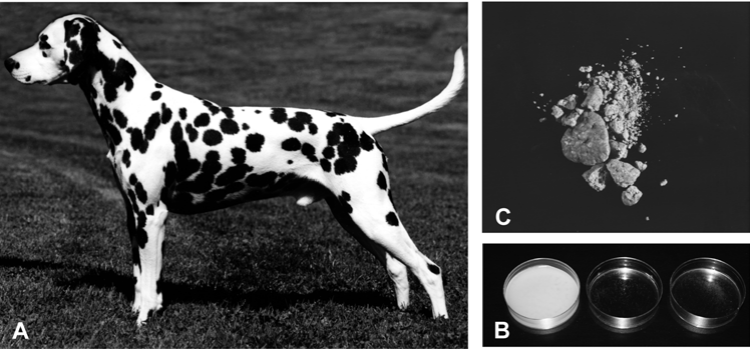
The Dalmatian phenotype. (A) The characteristic spotting pattern exhibited by the breed. (B) Urinary urate calculi removed from a Dalmatian. (C) Dalmatian urine crystallization: Dalmatian urine sample (left), urine from a heterozygous backcross dog (middle), and urine from a dog from an unaffected breed (right).

As in humans, all Dalmatian dogs have a defect in urinary metabolism that leads to excretion of uric acid rather than allantoin [11]. As a result, Dalmatians are predisposed to form urinary calculi composed of urate (Figure 1B). Hyperuricosuria in the Dalmatian is relatively easy to identify since Dalmatian urine forms a crystallized precipitate when cooled (Figure 1C). This trait was probably fixed in the breed through selection for a more distinctive spotting pattern [12],[13]. Dalmatian coat pattern involves mutations in at least three different spotting genes (Figure 1A). Dalmatians have a mutation for extreme white in the MITF gene [14] that leads to an all white coat. A dominant mutation, called T for ticking [15], is responsible for adding the pigmented spots to the white coat. Based on segregation analysis, the huu locus appears to be closely linked to a modifier of spot size [16].

In mammals that produce uric acid rather than allantoin, the level of uric acid in the blood is controlled by differences in production as well as differences in the amount that is excreted in the urine. In the kidney, uric acid is filtered by the glomerulus and then a portion is reabsorbed in the proximal tubules where it re-enters circulation. There are species-specific differences in the production of uric acid versus allantoin and the relative amounts of reabsorption and secretion in the proximal tubules, making the use of animal models in this area of research challenging [17].

Dogs and humans, unlike many other mammals, undergo bidirectional transport of urate along the nephron, which results in net reabsorption of urate from the glomerular filtrate. In Dalmatians, reabsorption is lost entirely and urate excretion equals or exceeds the glomerular filtration rate [18]. This change in uric acid excretion by the Dalmatian kidney is not secondary to hyperuricemia since non-Dalmatian dogs with artificially raised serum uric acid levels can only clear uric acid at ∼1/3 the rate of the Dalmatian [18]. Free-flow micropuncture experiments were used to demonstrate that in Dalmatian kidneys there is a deficiency of proximal tubular reabsorption of urate [19].

Although findings stated above implicate the kidney in Dalmatian huu, reciprocal liver and kidney transplant experiments between Dalmatian and non-Dalmatian dogs demonstrate that the liver is also important for the phenotype. Kidney transplants between normal dogs and Dalmatians only partially ameliorated the hyperuricosuria phenotype [20],[21]. However, Dalmatian hepatocyte transplants can correct the phenotype [20]. Therefore, a logical cause for the Dalmatian phenotype is a mutation in urate oxidase, similar to humans. However, Dalmatian liver homogenates are capable of oxidizing uric acid to allantoin and the urate oxidase gene was excluded genetically [3]. The Dalmatian phenotype could also be explained by a generalized defect of urate transport since liver slices were not capable of converting uric acid to allantoin [4]. Dalmatian dog erythrocytes have been shown to transport urate normally, demonstrating that Dalmatians do not have a generalized defect in urate transport [22]. Although Dalmatians have functional urate oxidase activity in their livers, they effectively have a similar phenotype to humans and great apes since they cannot transport urate into the liver for degradation. The Dalmatian phenotype can be summarized as a hepatic and renal urate transport defect which leads to hyperuricosuria and relative (compared to other dogs) hyperuricemia.

The discovery of various proteins that transport urate has shed some light on the control of serum and urine uric acid levels [23]. In the kidney, urate is transported across the apical membrane of the proximal tubules and then across the basolateral membrane before re-entering circulation. In humans, the transporter that reabsorbs urate across the apical membrane in the proximal tubule is Urat1, or SLC22A12 [24], which is expressed exclusively in the kidney. Mutations in SLC22A12 are thought to be the major cause of idiopathic renal hypouricemia in humans [24],[25], which is also called “Dalmatian hypouricemia” since people with this disorder spill uric acid into their urine resulting in a phenotype similar to the Dalmatian dogs [26]. In addition, recent work has shown an association of SLC2A9 with serum uric acid levels in several different populations [27],[28]. Variants in the non-coding region of SLC2A9 are associated with gout and uric acid levels in several human populations [29]–[31]. SLC2A9 has been shown by *Xenopus* oocyte experiments to transport uric acid [30]. In humans and mice it is expressed in liver and kidney [32],[33]. In particular, in humans SLC2A9 isoforms have been localized to both the apical and basolateral membrane of the proximal tubules, allowing the possibility that SLC2A9 influences serum uric acid levels by transport in the kidney [34].

All Dalmatians are homozygous for huu, which is inherited as a simple autosomal recessive trait as demonstrated by crosses performed between Dalmatians and other breeds of dogs [12],[13]. In order to identify the causative gene, an interbreed backcross (Dalmatian×Pointer) was developed which introduced the wildtype version of the huu gene into the Dalmatian breed while maintaining the breed characteristics of the Dalmatian. Based on linkage analysis using this cross (LOD 6.55), huu localizes to CFA03, excluding SLC22A12 as a candidate [35]. Using recombination breakpoints in the interbreed backcross and taking advantage of the homozygosity within the Dalmatian breed for huu, a critical interval containing four candidate genes was defined. Microsatellite markers within this interval gave LOD scores over 17 for linkage to huu. A candidate causal missense mutation (C188F) was identified within a highly conserved transmembrane (TM5) of the 12 transmembrane transporter protein, SLC2A9. The missense mutation is homozygous in all Dalmatians tested (247) as well as in hyperuricosuric dogs of other breeds.

## Results

**Figure 2:**
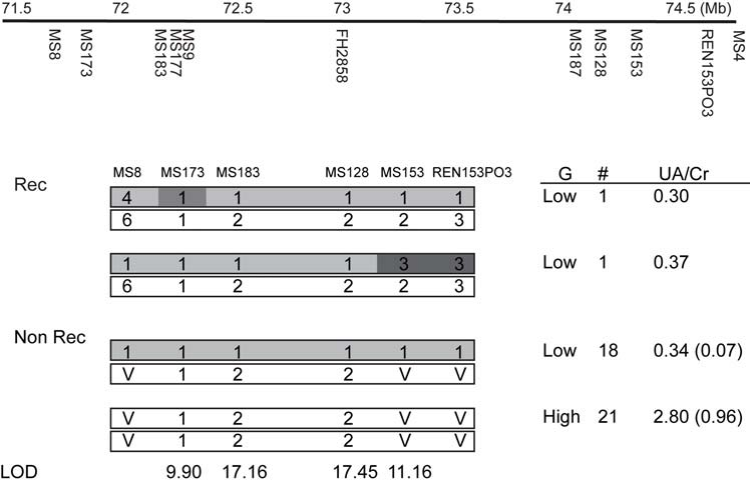
Fine structure mapping of the huu locus. (A) Physical map showing the location of the microsatellite markers used to map huu on CFA03. Microsatellite markers are listed below the line, and distances in Mb are shown above the line. (B) The haplotypes of recombinant (Rec) and non recombinant (Non Rec) backcross dogs are depicted with Dalmatian haplotypes in white and the Pointer haplotype in grey. A “V” indicates that the alleles were variable in Dalmatians. The first recombinant was not informative for MS173. The recombinants localized huu between MS8 and MS153. The maximum LOD score calculated for each microsatellite for all members of the family is shown at the bottom of the figure. The uric acid to creatinine ratio (UA/Cr), with the standard deviation in parenthesis, the number of dogs in each class (#), and the inferred huu genotype (G) are shown for dogs born since 2006 (28). The range of Uric acid/creatinine ratios for the low uric acid dogs is 0.23 to 0.47, and the range for high uric acid dogs is 1.2 to 4.0.

Linkage analysis using a Dalmatian×Pointer backcross family localized Dalmatian huu to CFA03. Haplotype analysis defined a 3.3 Mb critical interval (CFA03 72,063,073–75,355,028 Mb), estimated to contain 24 candidate genes [35]. Additional backcross dogs were genotyped with the microsatellites used for haplotype analysis and with new microsatellites mined from the critical region on CFA03. The full pedigree of all the dogs used for this analysis is shown in Figure S1. Urine uric acid/creatinine ratios were used to categorize the dogs' genotypes at huu. LOD scores were determined for a subset of these markers. Two markers within the critical interval defined by recombination breakpoints gave LOD scores over 17, further confirming the linkage to this region. Recombination events in two dogs narrowed the critical interval to a 2.5 Mb region containing 19 candidate genes (CFA03; 71,796,048–74,348,350 Mb) (Figure 2).

**Figure 3:**
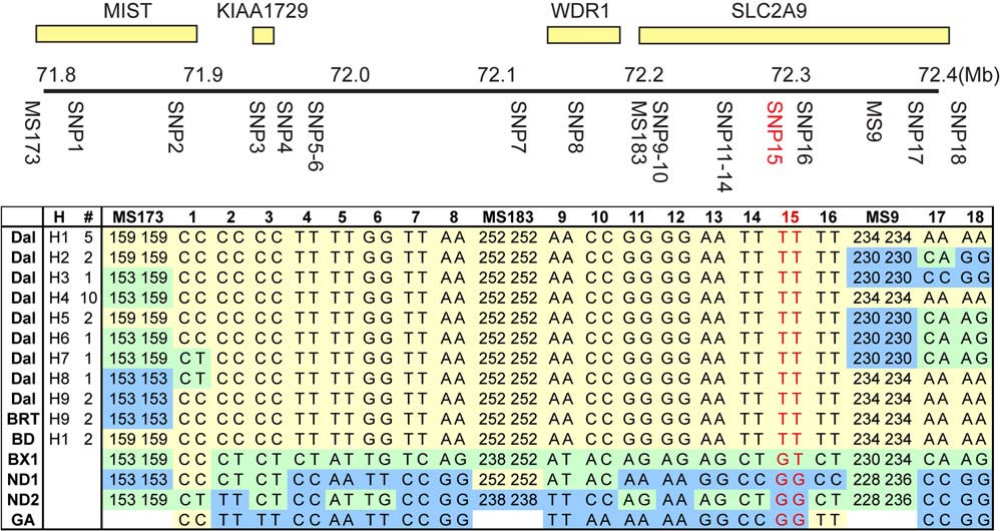
Homozygosity mapping of the huu locus. The four genes located in this interval are presented at the top of the figure. A region of CFA03 with distances in Mb above the line and marker locations below the line are shown. Genotypes across this region are shown in the boxed area. The breed is listed in the first column using the following abbreviations: Dal, Dalmatian; BRT, Black Russian Terrier; BD, Bulldog; BX1, heterozygous backcross dog; ND1 and ND2, mixed breed dogs; GA, genome assembly Boxer sequence. The different haplotypes are named in the second column, and the number of times that they were observed is shown in the third column. SNP15, shown in red, is the candidate causal mutation in exon 5 of SLC2A9. SNP9 and SNP10 are the promoter mutations in variant O. The common Dalmatian haplotype is shown in yellow, the non-Dalmatian genotype is shown in blue, and heterozygous genotypes are shown in green. Microsatellite allele sizes are absent for the genome assembly Boxer (GA) since DNA was not available for direct testing of the microsatellites.

Since Dalmatians are fixed for huu, it was expected that an area of homozygosity around the huu locus would be identified. Blocks of linkage disequilibrium (LD) in purebred dogs extend between several megabases in rare breeds with small population sizes to several kilobases in popular breeds with larger population sizes [14],[36]. Dalmatians have a moderate population size, so the homozygous region surrounding huu should be smaller than 2.5 Mb. Microsatellites mined from the canine genome and located ∼100 Kb apart were genotyped in the backcross dogs. Markers that were homozygous in huu/huu individuals and heterozygous in huu/+ individuals were typed in 24 unrelated Dalmatians to verify that the region of homozygosity is not the result of familial linkage disequilibrium. Haplotypes were constructed to rule out the possibility of more than a single ancestral mutation. Regions of homozygosity were found between ms173 and ms9 (CFA03; 71,796,048–72,363,187) and ms187 and ms128 (CFA03; 74,183,928–74,245,743). Eighteen SNPs, extending across 2.5 Mb, were genotyped in 24 Dalmatians, a wild-type Labrador Retriever and a huu/+ backcross dog. The results excluded the area between ms187 and ms128 and a total of 13 SNPs spanning ∼333 Kb confirmed the homozygous region between ms173 and ms9 in the 24 Dalmatians (Figure 3). These SNPs are also heterozygous in the huu/+ backcross dog, polymorphic in two unaffected dogs (ND1 and ND2) and the Boxer genome assembly sequence.

Four candidate genes were identified in the region of homozygosity, LOC488823 (similar to mast cell immunoreceptor signal transducer – MIST), LOC479092 (zinc finger protein 518B, KIAA1729), LOC611070 (similar to WD-repeat protein 1 – WDR1) and LOC479093 (similar to solute carrier family 2, member 9 protein, isoform 1 – SLC2A9) (Figure 3). RT-PCR established that all four genes were expressed in canine liver and kidney. Since all the candidates were expressed in the appropriate organs, all four genes were sequenced from Dalmatian and non-Dalmatian liver cDNA samples and the untranslated regions (UTRs) were determined by 5′ and 3′ RACE (Genbank EU371511–EU371515).

The 5′UTR and exons 1–7 of MIST were outside of the LD region and were not pursued. A single silent mutation was identified in exon 16 of the MIST gene (T951C). Three silent mutations were found in the single exon of the canine KIAA1729 gene (T932C, C2480T, G3092A) and a 33 bp insertion/deletion was discovered in the 3′UTR. Both alleles of these three SNPs and the insertion/deletion were seen in unaffected non-Dalmatian dogs. WDR1 was sequenced in liver cDNA and genomic DNA from a Dalmatian and a non-Dalmatian. A single SNP was found in intron 9 that does not affect a splice site. Although SLC2A9 did not have an assigned function related to uric acid metabolism at the time this work was performed, it was considered to be the most promising candidate since it is a transporter protein.

**Figure 4:**
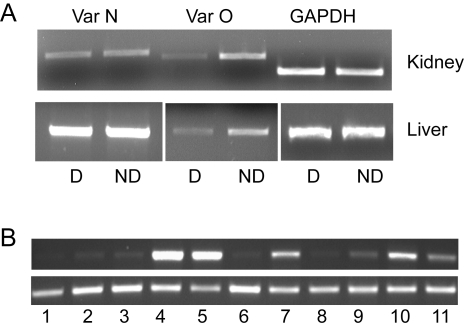
Tissue and isoform expression of canine SLC2A9. (A) Tissue expression by RT-PCR of canine SLC2A9 variant O, variant N, and GAPDH. D is liver and kidney RNA isolated from a Dalmatian, and ND is from a Newfoundland×Border Collie cross. (B) Tissue expression by RT-PCR of canine SLC2A9 on top and the GAPDH control on the bottom. Tissue samples from left to right are: 1 cerebellum, 2 cerebral cortex, 3 heart, 4 kidney, 5 liver, 6 skeletal muscle, 7 skin, 8 spinal cord, 9 spleen, 10 testis, 11 thymus.

A discrepancy in SLC2A9 exon annotation between NCBI and the UC Santa Cruz genome browser was addressed by 5′ RACE. Two SLC2A9 exon 1 variants were found in a Dalmatian and a wild-type Golden Retriever, variant O (CFA03:72,222,637–72,416,753) and variant N (CFA03: 72,227,605–72,416,753). The difference between the two variants lies in the first 28 amino acids of the N terminus, similar to known mouse and human variants. Both transcripts were shown to be expressed in canine liver and kidney by RT-PCR. Expression differences were observed for variant O between Dalmatian and non-Dalmatian in both liver and kidney (equivalent to isoform 2 in human) but not for variant N (Figure 4A). Expression in the Dalmatian samples was ∼50% of non-Dalmatian levels in both tissues. SLC2A9 expression was further evaluated by RT-PCR using primers that amplify both transcripts in 11 different tissues from an unaffected Beagle. The highest levels of expression were observed in the kidney and liver (Figure 4B).

**Figure 5:**
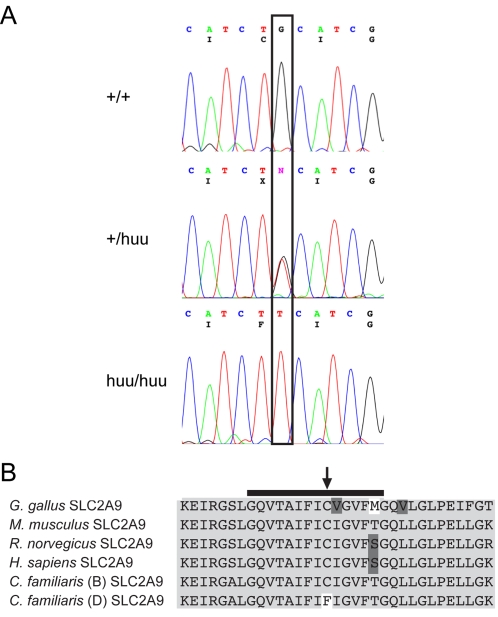
Sequence and expression of canine SLC2A9. (A) The electropherograms for the SLC2A9 sequence from a non-Dalmatian dog (+/+), heterozygous backcross dog (+/huu), and a Dalmatian dog (huu/huu) are shown. The nucleotide sequence is given, with the amino acid sequence presented below. The box shows the location of the mutation. (B) Protein sequence alignment of SLC2A9 around the exon 5 missense mutation from Dalmatians. The altered SLC2A9 amino acid is indicated with a vertical arrow. The horizontal line indicates the fifth transmembrane domain of the protein. The *C. familiaris* sequence is shown for Boxer (B) and Dalmatian (D).

Sequencing of the SLC2A9 gene was performed on canine liver cDNA as well as genomic DNA so that intron/exon boundaries and promoter regions could be evaluated. SLC2A9 coding exons 2–12, as well as the exon-intron boundaries, were sequenced in a Dalmatian and a Labrador Retriever. Six SNPs were discovered in the SLC2A9 sequence. Two are exonic; exon 5 G563T;Cys188Phe (nucleotide and amino acid numbering are reported with reference to variant N) and exon 11 G1303A;Val435Ile (nucleotide and amino acid numbering are reported with reference to variant N), two are located 99 and 101 bp 5′ to the start codon of variant O. SNPs were also identified in introns 1 and 10. None of the intronic SNPs are within or near conserved splice-site elements. The SLC2A9 exon 11 G1303A SNP is polymorphic in the 24 unrelated Dalmatians tested, consistent with its genomic location outside of the region homozygous in Dalmatians (SNP17, Figure 3). The polymorphisms located 5′ to the start codon were tested in a panel of DNA samples from 15 unaffected dogs from various different breeds. Both SNPs are polymorphic in unaffected non-Dalmatian dogs, displaying both the Dalmatian haplotype (A–C) and other combinations. These SNPs are always fixed in affected Dalmatian dogs (SNP9-10, Figure 3). Primary sequence data from the exon 5 SNP is shown in Figure 5A.

The two non-synonymous SNPs found in SLC2A9 coding sequence were tested using the PANTHER program (http://www.pantherdb.org/tools/), which assigned the Cys188Phe substitution a score of −4.047 (scores range between 0 to −10, −10 indicating the most deleterious) with a probability that it is a deleterious substitution of 0.74 [37]. The Val435Ile variation was not given a prediction since the substitution is not conserved in other species and unlikely to be deleterious. The SIFT program also scored the Cys188Phe missense mutation as deleterious with a probability of 0.01, where any probability below 0.05 is considered deleterious [38]. In order to evaluate the degree of protein conservation in the region flanking the candidate SNP, SLC2A9 protein sequences were compared between chicken, mouse, rat, human, Boxer and Dalmatian (Figure 4B). This region of the protein has a high degree of identity across mammals. The variant O promoter SNPs (located 99 and 101 base pairs upstream of the initiator methionine) were analyzed for transcription factor binding site differences and the SNPs are predicted to disrupt binding sites for DeltaE, AML-1a, S8 and Cre-BP in the Dalmatian version.

The SLC2A9 exon 5 C188F amino acid substitution was further tested in 247 Dalmatians and 378 non-Dalmatian dogs from 58 different breeds. It was homozygous in all Dalmatians tested and only the wildtype allele was present in the non-Dalmatian dogs.

Individual dogs from non-Dalmatian breeds are known to form urate calculi and have been diagnosed with huu [39],[40]. Two Dogs from two of these breeds, Bulldogs and Black Russian Terriers, that had formed urinary urate calculi were tested for the SLC2A9 exon 5 G563T;Cys188Phe missense mutation (SNP 15 in Figure 3) and the variant O promoter SNPS (SNP 9, 10 in Figure 3) and found to be homozygous. These four dogs shared the Dalmatian haplotype across the homozygous interval defined during the positional cloning of the huu locus (Figure 3).

## Discussion

The Dalmatian dog exhibits hyperuricosuria and relative hyperuricemia due to a defect of urate transport in the liver and kidney. Positional cloning of the huu locus using an interbreed backcross, as well as homozygosity within the Dalmatian breed, has identified SLC2A9 as the cause of the change in uric acid handling by this dog breed. Unrelated breeds of dog with hyperuricosuria or urate stone disease share the same haplotype as Dalmatian dogs, providing compelling evidence that this is the gene responsible. This result was somewhat unexpected because SLC2A9 was classified as a member of the large glucose transporter family and did not have an assigned function with respect to urate transport until recently.

SLC2A9 is classified as a part of the large glucose transporter family based on amino acid sequence identity of 44% and 38% to Glut5 and Glut1, respectively [32]. SLC2A9 has been localized to the cell surface in humans [34]. It contains 12 transmembrane domains and, based on homology to other glucose transporters, the central channel is essentially formed by helices 2, 4, 5, 7, 8, and 10 [41]. The Cys188Phe amino acid change occurs within a highly conserved residue located within the fifth transmembrane domain of the protein. Although a cysteine to phenylalanine amino acid change is not expected to disrupt the localization of the alpha helix to the transmembrane, this change could disrupt the proper function of the protein by altering the pore. Precedent for single amino acid changes altering substrate specificity has been shown for a number of the SLC2A transporters [42].

Expression differences between Dalmatian and non-Dalmatian samples were observed for one of the isoforms (O) of the gene (Figure 4). SNPs in the promoter region of this variant were identified and were fixed in the Dalmatian breed. These same SNPs were also identified in non-Dalmatians without hyperuricosuria. Therefore, these SNPs alone are not sufficient to confer the phenotype but along with the missense mutation they may contribute to the expression of the phenotype. It remains to be determined if there are variant O transcript differences in the general dog population and if the SNPs identified in this work are responsible for the differences in the level of variant O expression. In humans, the equivalent isoform to variant O is expressed on the apical surface of the proximal tubules [34]. Reduction in the amount of the protein combined with a deficiency in the protein itself may contribute to the decreased conversion to allantoin by the liver as well as the increased excretion of uric acid in these dogs. SLC2A9 tissue expression in dogs is similar to that observed in humans and mice. Expression in humans is highest in kidney, liver and placenta [32]. In humans, isoform 1 has been localized within polarized canine kidney cells to the basolateral membrane of the proximal tubules while isoform 2 is localized to the apical side [34]. The tissue and cellular localization of SLC2A9 is consistent with the Dalmatian huu phenotype and with SLC2A9 having a role in urate transport in both the liver and kidney.

Mutations in SLC2A9 will likely have important consequences for a number of different disorders of uric acid homeostasis in people. Significant alterations of SLC2A9 could cause primary renal hypouricemia in people similar to SLC22A12 mutations since genetic heterogeneity exists for this disorder [43]–[45]. Mutations in SLC2A9 could also cause cases of primary gout by significantly altering the serum uric acid concentration. In addition, more subtle changes could alter serum uric acid levels by changing the amount of uric acid excreted in the urine. There is evidence that polymorphisms in SLC22A12 are capable of increasing serum uric acid levels in Japanese populations [46]. A significant association of SLC2A9 to serum uric acid levels was recently reported among Caucasian individuals [28] and among Sardinian and Chianti cohorts [27]. Two other papers were recently published on the importance of the SLC2A9 gene to urate transport in humans. The first demonstrated that human SLC2A9 is a high capacity, low affinity uric acid transporter and that genetic variants are associated with gout; however causative mutations were not determined [30]. The second paper documented a strong association between SLC2A9 and uric acid levels in cohorts of German and Austrian nationalities as well as a correlation between RNA expression levels of this gene and serum uric acid levels [29]. Thus, strong evidence exists that SLC2A9 functions as a urate transporter in humans, and likely in dogs.

In addition to the potential influence with respect to human uric acid disorders, the present studies also impact our understanding of the history of this defect in dogs. The story of hyperuricosuria in dogs started in 1916 when Benedict first recognized the similarity of uric acid defects in the Dalmatian dog and people [11]. However, the origin of this defect may have preceded the development of the Dalmatian breed. The present studies show that the same genetic mutation is present in Bulldogs and Black Russian Terriers, breeds that are not known to be closely related to the Dalmatian. It appears that affected individuals from these breeds share the same haplotype as the Dalmatian, indicating that the mutation is identical by descent between these breeds. The mutation must be quite old since it would have to predate breed formation; however, additional evaluation of the extended haplotype in all three breeds is necessary to estimate an actual age. Alternatively, although unlikely, the mutation could have been introduced into these other breeds by crosses between breeds. Although Dalmatians are fixed for hyperuricosuria, this is not true of the other breeds. Therefore, genetic testing and selection in those breeds can eliminate the disease. Within the Dalmatian breed, the possibility exists for correction of this defect by the introduction of unaffected Dalmatian×Pointer backcross dogs into the purebred gene pool. These dogs are currently registered with the United Kennel Club in the United States. The disease allele probably became homozygous in modern Dalmatians through selection for more distinctive spots. However, most low uric acid excreting backcross dogs have acceptably sized spots (according to the breed standard), allowing breeders the unique opportunity to correct a fixed genetic defect while maintaining the breed characteristic that may ultimately be responsible for its fixation.

Two independent lines of evidence from different species point to the key role of SLC2A9 in urate transport; the Dalmatian uric acid phenotype itself and genome wide association studies linking SLC2A9 to uric acid levels in people along with direct uric acid transport data. There are many questions to be answered about the role of SLC2A9 in urate homeostasis and its other transport functions. In dogs and other mammals with endogenously low serum uric acid, SLC2A9 may normally play a different transport role than in people where it appears to have an important function in uric acid transport. Since degradation of uric acid to allantoin does not occur in humans and great apes, SLC2A9 may play a different role in transport in the liver as compared to those species that excrete allantoin. The positional cloning of the hyperuricosuria locus in the Dalmatian dog has provided a compelling new avenue of investigation toward a better understanding of urate transport in mammals and successfully completes a story started in 1916 when Benedict first recognized the similarity of the uric acid defect in the Dalmatian dog and people.

## Materials and Methods

DNA samples from backcross dogs were acquired as previously described [35]. The full pedigree of the dogs used in this analysis is shown in Figure S1. Blood, buccal swab and DNA samples from Dalmatians and non-Dalmatian dogs were obtained from patients of the Veterinary Medical Teaching Hospital at UC Davis, the UC Davis Veterinary Genetics Laboratory, Dr. Gary Johnson at the University of Missouri, Columbia, and from private owners. The use of animals in this research was approved by the University of California, Davis animal care and use committee (protocol #11962). Urine uric acid and creatinine was measured in 3–7 week old puppies as previously described [13].

LOD scores were calculated as previously described [35]. Primers for microsatellites spaced on average 100 Kb apart in the 3.3 Mb candidate region of CFA03 were identified using the May 2005 CanFam2.0 sequence assembly on the UCSC genome browser and designed within sequence flanking each repeat using the Primer3 program (Table S1) [47].

Eight SNPs, which are part of the Affymetrix canine SNP array, were chosen because they are polymorphic in the general dog population. Additional SNPs were mined from the canine genome sequence or identified during sequencing of the candidate genes. Primers for SNPs (Table S2) were designed as described above. SNP and microsatellite sequences were PCR amplified and genotyped in the backcross dogs and only informative markers for this family were then genotyped in 24 unrelated purebred Dalmatians.

Human mRNA sequences were obtained from GenBank for each of the candidate genes (MIST NM_052964.1, KIAA1729 NM_053042, WDR1 NM_005112.4, SLC2A9 AF210317). These sequences were compared to the UCSC Genome Browser annotation of the canine genome using the BLAT function to obtain canine sequence for each gene. The Primer3 program was used to design primers for the canine sequences (Table S3). PCR reactions, genotyping and sequencing were done as previously described [35]. Sequences were visualized using Chromas2 (Technelysium, Tewantin, QLD, Australia) and analyzed with Vector NTI software (Informax, Frederick, MD, USA). TFsearch Version 1.3 was used to analyze the Variant O promoter SNPs affect on transcription factor binding sites.

5′ and 3′ RACE were performed for 3 of the 4 candidate genes. RACE primers (Table S3) were designed as described above and RACE products amplified with the SMART RACE cDNA Amplification Kit (Clontech, Mountain View, CA, USA) and cloned using the TOPO TA Cloning kit (pCR2.1-TOPO vector) with One Shot TOP10 Chemically Competent E. coli (Invitrogen, Carlsbad, CA, USA). Products were isolated with the Qiaprep Spin Miniprep kit (Qiagen, Valencia, CA, USA) and sequenced as described above. Genbank accession numbers for the transcripts are EU371511–EU371515. All genomic locations given in the text are based on the May 2005 CanFAm2.0 genome assembly and are viewed using the UCSC genome browser.

RNA was isolated from kidney and liver samples with the Micro-FastTrack 2.0 mRNA isolation kit (Invitrogen, Carlsbad, CA, USA). cDNA was synthesized with the SuperScript III First-Strand Synthesis System for RT-PCR (Invitrogen, Carlsbad, CA, USA). Expression was evaluated for LOC479092, LOC611070 and both variants of LOC479093 from liver and kidney from Dalmatians and unaffected Newfoundland×Border Collie crosses using primers SLC5′UTREx1VarNF and SLCR1 for variant N and primers IntF-Ex1 and SLCR1 for variant O. The expression of LOC479093 was also evaluated in an array of tissues from an unaffected Beagle (cerebellum, cerebral cortex, heart, kidney, liver, skeletal muscle, skin, spinal cord, spleen, testis and thymus) using primers IntF5 and SLCdnaR. RNAs for these tissues were acquired from Zyagen (San Diego, CA, USA) and cDNA synthesized as described. GAPDH was amplified (F primer-5′AAGATTGTCAGCAATGCCTCC3′, R primer -5′CCAGGAAATGAGCTTGACAAA3′) in these tissues to ensure that equivalent amounts of cDNA were added.

SLC2A9 genotypes were determined by a restriction fragment length polymorphism (RFLP) assay. PCR products were produced as described previously using an unlabeled forward primer, 5′-TGCTTCTCTGAAATTTACCTCCA – 3′ and a fluorescently labeled reverse primer, 5′-6FAM-CGAGAGGATGGTATACGGTGA -3′ (Applied Biosystems, Foster City, CA). Products were then digested with the enzyme HpyCH4V (New England Biolabs, Ipswitch, MA) for 1 hour at 37°C. Digestions were analyzed on an ABI 3100 Genetic Analyzer with GeneScan 400HD Rox size standard. A 79 base pair unlabeled product is generated from the 440 bp product by a control cut site. The A allele produces a labeled 361 base pair product and the G allele produces a labeled 106 base pair product.

## Supporting Information

Table S1 Primers for microsatellite markers used to define the Huu region.
(0.03 MB XLS)

Table S2 Primers for SNPs used to define the Huu region.
(0.03 MB XLS)

Table S3 Candidate gene primers.
(0.03 MB XLS)

Figure S1 Numbers indicate that DNA was available; individual 3 is placed twice in the pedigree for clarity, individuals numbered 39 and higher were genotyped and phenotyped after Safra et al. (2006). Standard genetic symbols are used for sex and affectation status. The single male Pointer used in this pedigree is shown at the top; all other dogs were purebred Dalmatians or backcross progeny.
(12.17 MB TIF)
