# Supplementary figures and images for: Mutations in the SLC2A9 Gene Cause Hyperuricosuria and Hyperuricemia in the Dog
Source: PLoS Genet. 2008 Nov 7;4(11):e1000246. doi: 10.1371/journal.pgen.1000246 (PMC2573870; doi:10.1371/journal.pgen.1000246)

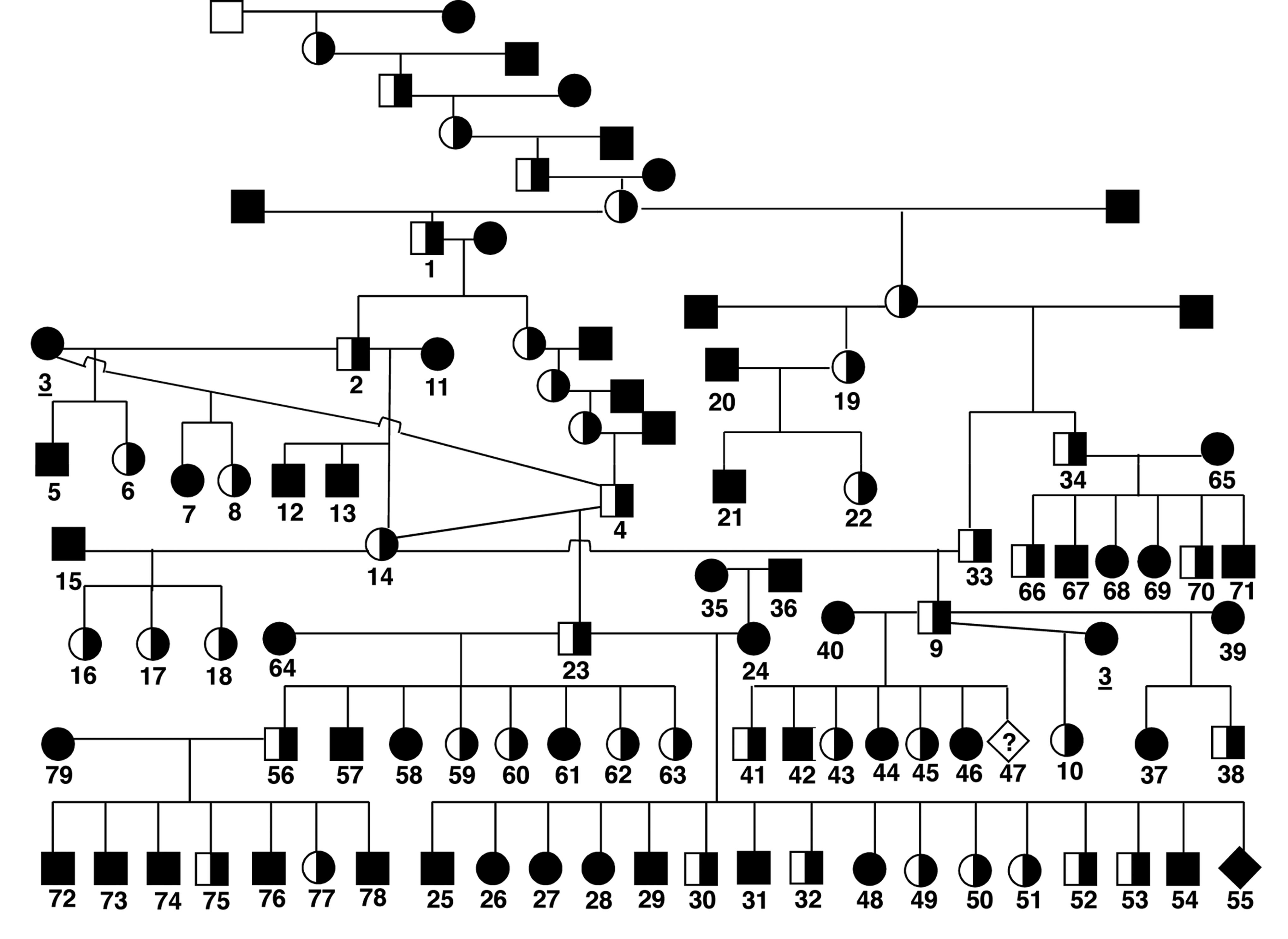

Supplement: Figure S1 — Numbers indicate that DNA was available; individual 3 is placed twice in the pedigree for clarity, individuals numbered 39 and higher were genotyped and phenotyped after Safra et al. (2006). Standard genetic symbols are used for sex and affectation status. The single male Pointer used in this pedigree is shown at the top; all other dogs were purebred Dalmatians or backcross progeny. (12.17 MB TIF) [file pgen.1000246.s004.tif]
